# Artificial intelligence-generated targets and inter-observer variation in online adaptive radiotherapy of bladder cancer

**DOI:** 10.1016/j.phro.2024.100640

**Published:** 2024-09-01

**Authors:** Lina M. Åström, Patrik Sibolt, Hannah Chamberlin, Eva Serup-Hansen, Claus E. Andersen, Marcel van Herk, Lene S. Mouritsen, Marianne C. Aznar, Claus P. Behrens

**Affiliations:** a Department of Oncology, Copenhagen University Hospital – Herlev and Gentofte, Copenhagen, Denmark; b Department of Health Technology, Technical University of Denmark, Roskilde, Denmark; c Division of Cancer Sciences, School of Medical Sciences, Faculty of Biology, Medicine and Health, University of Manchester, Manchester, United Kingdom

## Abstract

**Background and purpose:**

Daily target re-delineation in online adaptive radiotherapy (oART) introduces uncertainty. The aim of this study was to evaluate artificial intelligence (AI) generated contours and inter-observer target variation among radiotherapy technicians in cone-beam CT (CBCT) guided oART of bladder cancer.

**Materials and methods:**

For each of 10 consecutive patients treated with oART for bladder cancer, one CBCT was randomly selected and retrospectively included. The bladder (CTV-T) was AI-segmented (CTV-T_AI_). Seven radiotherapy technicians independently reviewed and edited CTV-T_AI_, generating CTV-T_ADP_. Contours were benchmarked against a ground truth contour (CTV-T_GT_) delineated blindly from scratch. CTV-T_ADP_ and CTV-T_AI_ were compared to CTV-T_GT_ using volume, dice similarity coefficient, and bidirectional local distance. Dose coverage (D_99%_>95 %) of CTV-T_GT_ was evaluated for treatment plans optimized for CTV-T_AI_ and CTV-T_ADP_ with clinical margins. Inter-observer variation among CTV-T_ADP_ was assessed using coefficient of variation and generalized conformity index.

**Results:**

CTV-T_GT_ ranged from 48.7 cm^3^ to 211.6 cm^3^. The median [range] volume difference was 4.5 [−17.8, 42.4] cm^3^ for CTV-T_ADP_ and −15.5 [−54.2, 4.3] cm^3^ for CTV-T_AI_, compared to CTV-T_GT_. Corresponding dice similarity coefficients were 0.87 [0.71, 0.95] and 0.84 [0.64, 0.95]. CTV-T_GT_ was adequately covered in 68/70 plans optimized on CTV-T_ADP_ and in 6/10 plans optimized on CTV-T_AI_ with clinical margins. The median [range] coefficient of variation was 0.08 [0.05, 0.11] and generalized conformity index was 0.78 [0.71, 0.88] among CTV-T_ADP_.

**Conclusions:**

Target re-delineation in CBCT-guided oART of bladder cancer demonstrated non-isotropic inter-observer variation. Manual adjustment of AI-generated contours was necessary to cover ground truth targets.

## Highlights

• Small inter-observer variation in targets manually edited by radiotherapy technicians.
• Delineation uncertainty was non-isotropic.
• Artificial intelligence algorithms consistently underestimated the bladder target.
• Manual adjustment of artificial intelligent generated contours was necessary to ensure target coverage.

## 1 Introduction

Target delineation variation is a major challenge in radiotherapy. Together with anatomical variations, it is one of the largest uncertainties to consider during treatment planning [1], [2]. Traditionally, in the margin concept first introduced by van Herk *et al*., the delineation uncertainty is defined as a systematic component that leads to a displacement of the dose distribution with respect to the clinical target volume (CTV) [3]. While this is true for conventional non-adaptive image-guided radiotherapy, where targets are delineated once prior to treatment start, it may become more of a random uncertainty in online adaptive radiotherapy (oART), where targets are re-delineated daily, often by different observers. However, it may still have a systematic component, e.g., due to influence or guidance by reference contours in the re-delineation procedure.

The use of oART has vastly increased during the last decade, through the introduction of advanced systems, some of which utilizing artificial intelligence (AI) [4], [5]. Several studies have demonstrated the clinical feasibility of cone-beam computed tomography (CBCT) guided oART for various disease sites, especially in the pelvis [6], [7], [8], [9], [10], [11]. When the treatment is completely re-optimized to the daily anatomy, any inter-fractional anatomical variations are considered, enabling the margins to be reduced. For bladder cancer, we have previously reported large margin reductions, resulting in sparing of organs at risk (OARs) [12], [13]. However, if not accounted for, uncertainties related to AI-generated contours and daily re-delineation of targets may have a negative effect on the clinical outcome.

The magnitude of the delineation uncertainty depends on several factors, such as image quality (modality, resolution, contrast, artifacts), observer (experience and educational background), use of delineation guidelines, format of contouring (from scratch, automatic segmentation, or propagation from a reference image), and time pressure (workload/throughput) [1], [14], [15], [16], [17], [18], [19]. Previous studies have investigated inter-observer CTV delineation variation for bladder cancer on CBCT [20], [21], but the contours were delineated by physicians without any time constraint. Since the anatomy within the pelvis is highly dynamic, time-efficient delineation is crucial in oART. Furthermore, to reduce the need for daily online support by physician and thus make oART clinically practicable, radiotherapy technicians are increasingly taking on responsibilities previously attributed to the physicians [22], [23]. The aim of this study was to assess inter-observer CTV variation in oART of bladder cancer, among radiotherapy technicians performing oART routinely. Additionally, the quality of AI-generated bladder contours and the need for editing them were evaluated. Contours were benchmarked against ground truth contours to assess the impact of identified discrepancies on dose coverage.

## 2 Materials and methods

### 2.1 Patients and observers

This retrospective study included 10 consecutive patients treated with curative-intended CBCT-guided oART for muscle-invasive bladder cancer between February 2020 and February 2021 (Supplementary Table S1). Patient cases included three males and seven females, one patient had a hip prosthesis, and three patients had a catheter. A total dose of 64 Gy in 32 fractions was prescribed to the primary planning target volume (PTV-T), according to Danish national and international guidelines [24], [25]. All patients were treated at an Ethos Therapy v1.1 treatment unit (Varian, a Siemens Healthineers Company). Further details on the treatment are included in a previous publication [12].

A total of seven adapters participated in this study. The adapters consisted of radiotherapy technicians (4 nurses and 3 radiographers) that had completed a one-year national training program in radiotherapy as well as a departmental training program in CBCT-guided oART and who routinely carried out oART clinically without daily support by physicians and/or physicists. The training program included anatomy and oART lectures by physicians and physicists, supervised and non-supervised offline delineations, and online supervised delineations. This study was approved by the department as part of the quality assurance program for CBCT-guided oART. All patients were anonymized during the conduction and analysis of the study.

### 2.2 Contouring guidelines and margins

Reference CT and magnetic resonance imaging (MRI) scans were acquired approximately one week prior to treatment start (scanning details in Supplementary Materials). The patients were instructed to have an empty bladder and rectum during reference scans and treatments. They were instructed to avoid drinking fluids two hours prior to both scans and treatments to reduce the intra-fractional anatomical variation.

The primary CTV (CTV-T) was defined as the outer wall of the bladder including any visible tumour as seen on CT, with possible extra-bladder extension caudally as guided by the reference MRI. When indicated according to Danish national guidelines [24], the CTV-T involved prostate and/or seminal vesicles.

PTV-T was generated by adding patient-specific anisotropic margins of 5–12 mm to CTV-T (Supplementary Table S1). Generally, the margins were 5 mm plus the maximum intra-fractional bladder variation as measured on pre- and post-adaptation CBCTs during two to four non-adaptive fractions prior to the start of oART. The intra-fractional bladder variation was measured in six directions (cranial, caudal, anterior, posterior, left, right) using anatomical landmarks (e.g., symphysis, femoral heads, and spinal column) as reference measuring points [12].

### 2.3 Inter-observer delineation variation

One session CBCT was randomly selected among the oART sessions in each of the 10 patients’ treatment courses and imported to an emulator with Ethos Treatment Management System v1.1. The CBCTs were acquired on Ethos Therapy v1.1 with the settings reported in Supplementary Table S1 and reconstructed with a slice thickness of 2 mm and a pixel size of 0.96 mm × 0.96 mm. Each adapter independently and blindly simulated one selected oART session per patient. In the oART session, the bladder was auto-contoured from scratch by integrated AI-algorithms, which followed the version of the system and did not learn based on input from the user (neither reference delineations nor previous fractions). The adapters reviewed and manually edited the AI-generated bladder contour, generating CTV-T_ADP_, within a clinical time frame, i.e., as if it was a clinical treatment (typically within 10 min [12]). Just as in the clinical workflow, the reference CT and -contours was available during the simulations [12]. An additional oART session was simulated for each patient without any edit of the AI-generated contours to generate CTV-T_AI_. In parallel, a ground truth CTV-T (CTV-T_GT_) was first delineated by a senior oncologist, then reviewed by an oART experienced medical physicist, and finally edited to a consensus contour, all blind to the adapter’s delineations but with reference CT and -delineations available. Contouring of CTV-T_GT_ was carried out in Eclipse treatment planning system v15.1 (Varian, a Siemens Healthineers Company) without any time constraint. If the prostate and seminal vesicles were included in the reference CTV-T, only the bladder part was included in CTV-T_ADP_, CTV-T_AI_, and CTV-T_GT_. Further details on Ethos and the oART workflow can be found elsewhere [4], [12].

### 2.4 Data analysis

All CTV-T_ADP_ and CTV-T_AI_ were compared to corresponding CTV-T_GT_ using volume, dice similarity coefficient (DSC), and bidirectional local distance (BLD). DSC was calculated in Eclipse and BLD was calculated using Python. BLD was calculated at each point on the surface of CTV-T_GT_ (p_GT_), by calculating: 1) the minimum absolute distance from p_GT_ to CTV-T_ADP_ or CTV-T_AI_, and 2) the minimum absolute distances from each point on the comparator surface to CTV-T_GT_ and selecting the largest of distances connected to p_GT_. BLD at p_GT_ was then defined as the maximum of 1) and 2) [26].

The dose covering 99 % of the CTV-T_GT_ and PTV-T_GT_ (D_99%_>60.8 Gy in accordance with departmental guidelines) was evaluated for treatment plans optimized on PTV-T_ADP_ as well as PTV-T_AI_, i.e., CTV-T_ADP_ and CTV-T_AI_ plus the clinical patient-specific CTV-T-to-PTV-T margins in Supplementary Table S1. PTV-T_GT_ was evaluated as coverage of a larger volume than CTV-T_GT_ was desired due to the large intra-fractional variations of the bladder [27], [28]. However, since PTV-T_GT_ accounted for several uncertainties and the dose coverage of it is sensitive to small differences between CTV-T_GT_ and CTV-T_ADP_ or CTV-T_AI_, complete coverage was not necessary nor expected. All plans were generated in Ethos as IMRT with 12 equidistant fields.

The inter-observer delineation variation was evaluated by calculating the generalized conformity index (CI_gen_) and coefficient of variation (CV) among CTV-T_ADP_, for each patient. CI_gen_ was defined as the ratio of the sum of all overlapping volumes between pairs of observers and the total volume of the same pair, i.e., CI_gen_=∑_pairs ij_|V_i_ ∩ V_j_| / ∑_pairs ij_|V_i_ ∪ V_j_| [29]. CV was calculated as the ratio between the standard deviation and the mean volume.

## 3 Results

**Fig. 1:**
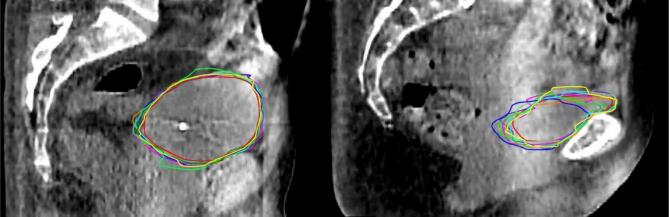
Sagittal view of CBCT with contours of CTV-T_GT_ (yellow), CTV-T_AI_ (red), CTV-T_ADP_ (remaining colours) for patient 1 (left) and 10 (right), with relatively low and high inter-observer delineation variation, respectively. CI_gen_ was 0.88 for patient 1 and 0.71 for patient 10. Both patients have given written consent to use of images and treatment plans. (For interpretation of the references to colour in this figure legend, the reader is referred to the web version of this article.)

**Fig. 2:**
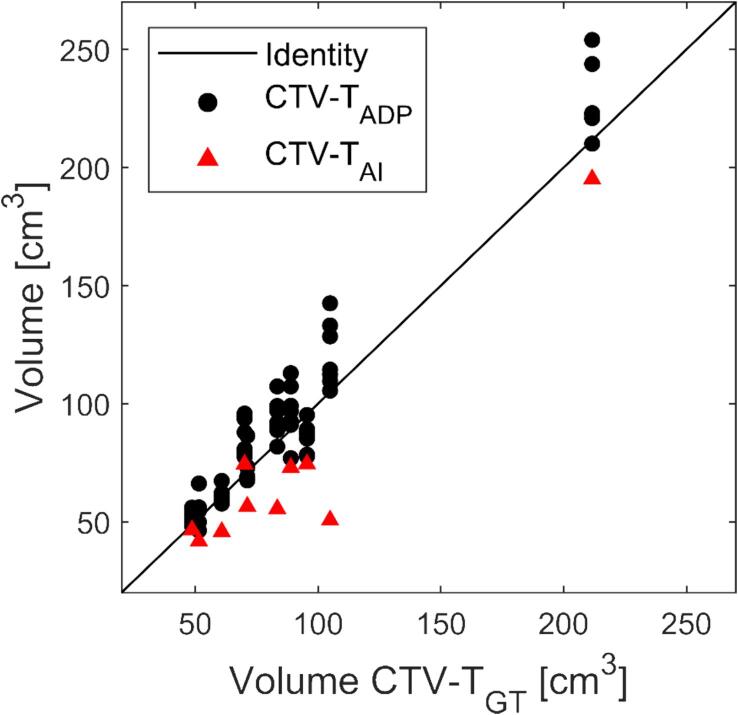
Identity plot with volume of CTV-T_ADP_ (black dots) and CTV-T_AI_ (red triangles) as a function of CTV-T_GT_ volume. From the left, the patients are in the following order: 3, 5, 2, 4, 6, 9, 7, 10, 8, and 1. (For interpretation of the references to colour in this figure legend, the reader is referred to the web version of this article.)

**Fig. 3:**
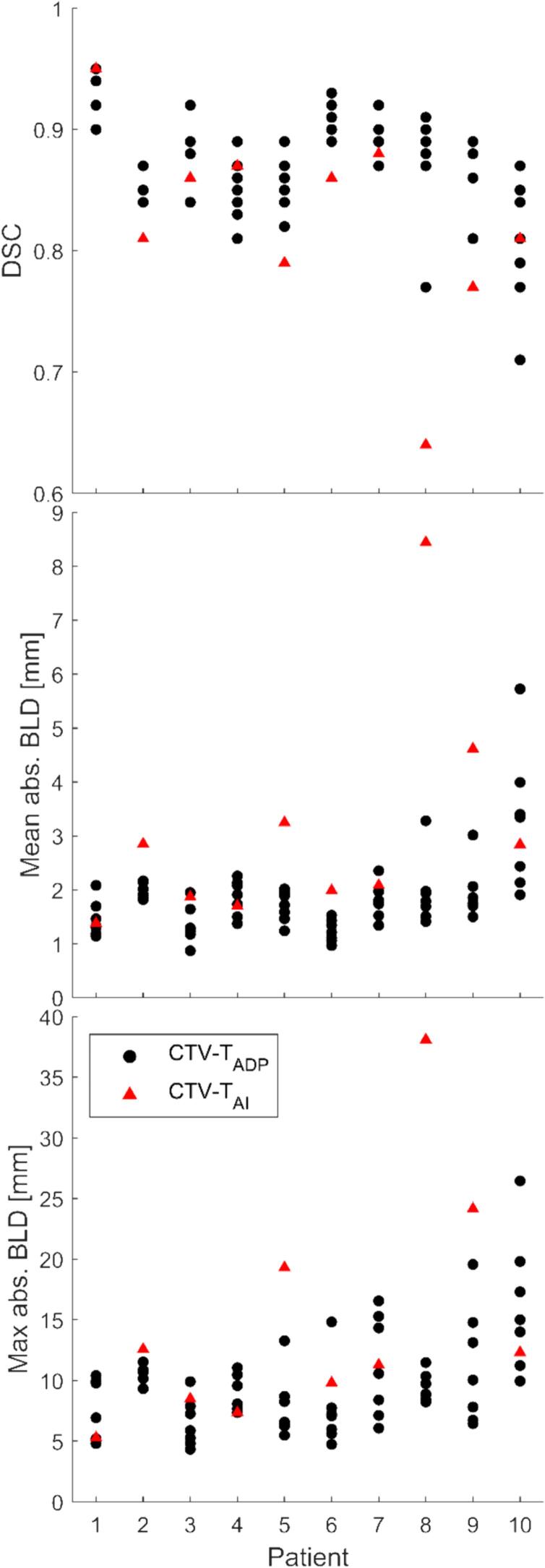
(Top) Dice Similarity Coefficient (DSC), (middle) mean absolute Bilateral Local Distance (BLD), and (bottom) maximum absolute BLD comparing CTV-T_ADP_ (black dots) and CTV-T_AI_ (red triangles) with CTV-T_GT_ for each patient. (For interpretation of the references to colour in this figure legend, the reader is referred to the web version of this article.)

**Fig. 4:**
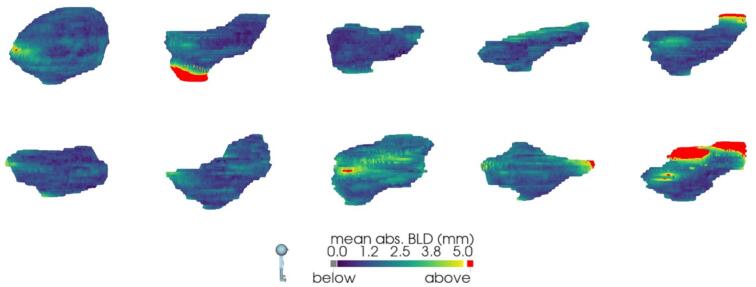
Viewed from the right side of the patient, i.e., anterior is to the right and posterior is to the left in each Fig. 2-D colour maps of mean of the absolute bidirectional local distance (BLD) across all seven adapters for patient 1–10, comparing CTV-T_GT_ and CTV-T_ADP_. The patients are ordered in a left–right, top–bottom manner. The colour scale goes from 0 mm to 5 mm, where values ≥ 5 mm are coloured in red. (For interpretation of the references to colour in this figure legend, the reader is referred to the web version of this article.)

Fig. 1 presents the nine contours (one CTV-T_GT_, one CTV-T_AI_, and seven CTV-T_ADP_) on two different patients: one with relatively low and another with relatively high inter-observer delineation variation. Over all 10 patients, the CTV-T_GT_ ranged from 48.7 cm^3^ to 211.6 cm^3^. The median [range] volume difference was 4.5 [−17.8, 42.4] cm^3^ for CTV-T_ADP_ and −15.5 [−54.2, 4.3] cm^3^ for CTV-T_AI_ (Fig. 2, Supplementary Table S1). This corresponds to relative mean [range] volume differences of 5.3 % [−18.6 %, 36.5 %] and −18.8 % [−51.7 %, 6.1 %], respectively. CTV-T_AI_ was smaller than all corresponding CTV-T_GT_ except one and all corresponding CTV-T_ADP_. The results for DSC and BLD comparing CTV-T_ADP_ and CTV-T_AI_ to CTV-T_GT_ are shown in Fig. 3. The median [range] DSC was 0.87 [0.71, 0.95] for CTV-T_ADP_ and 0.84 [0.64, 0.95] for CTV-T_AI_. Two-dimensional colour maps of the mean absolute BLD across all adapters at each point on CTV-T_GT_ (Fig. 4), demonstrated that the areas with largest deviation of CTV-T_ADP_ compared to CTV-T_GT_ were patient-dependent but primarily in cranial, caudal, anterior, and posterior directions. Studying the deviations for each CTV-T_ADP_ (Supplementary Figure S1), the deviations were not isotropic but concentrated to specific areas that varied among the patients. The BLD comparing CTV-T_AI_ and CTV-T_GT,_ varied over the volume and among the patients, but the largest absolute BLD was observed in cranial, caudal, and anterior direction (2-D maps are included in Supplementary Figure S2 and S3).

**Fig. 5:**
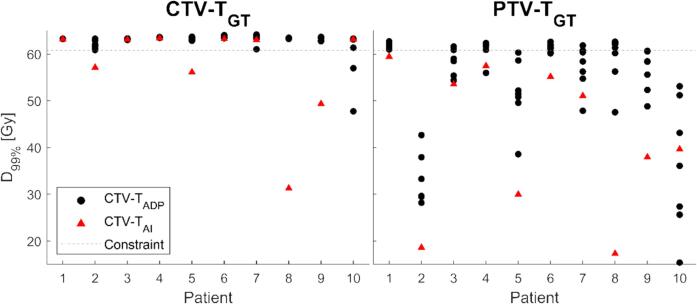
D_99%_ of CTV-T_GT_ (left) and PTV-T_GT_ (right) for plans optimized on CTV-T_ADP_ (black dots) and CTV-T_AI_ (red triangles) with clinical CTV-T-to-PTV-T margins. (For interpretation of the references to colour in this figure legend, the reader is referred to the web version of this article.)

Treatment plans optimized on PTV-T_ADP_ and PTV-T_AI_ did not cover (D_99%_<60.8 Gy) CTV-T_GT_ in 2/70 (patient 10) and 4/10 (patient 2, 5, 8, and 9) plans, respectively (Fig. 5). Dose coverage of CTV-T_GT_ was lacking in anterior and cranial directions for PTV-T_ADP_ plans and in cranial, caudal, and anterior directions for PTV-T_AI_ plans. PTV-T_GT_ was not covered in 45/70 and 10/10 plans, respectively (Fig. 5).

Among CTV-T_ADP_, the median [range] CI_gen_ was 0.78 [0.71, 0.88] and CV was 0.08 [0.05, 0.11]. Colour maps of two standard deviations of absolute BLD among CTV-T_ADP_ show that the largest disagreement among the adapters was in the cranial, anterior, and posterior directions (2D maps are included in Supplementary Figure S4).

## 4 Discussion

We present novel data on inter-observer target variation among adapters and quality of AI-generated contours in CBCT-guided oART of bladder cancer. Our results on CI_gen_ and CV among CTV-T_ADP_ are comparable to other studies on inter-observer target variation of bladder cancer among physicians on CBCT. Foroudi *et al.* [20] report a mean CI (defined as the ratio between overlapping volumes and the total volume) of 0.75 for four patients and four radiation oncologists, and Nishioka *et al.* [21] report a mean CI_gen_ of 0.81 and a mean CV of 0.08 for ten patients and five radiation oncologists. Studying the colour maps in Supplementary Figure S4, the areas with largest disagreement among CTV-T_ADP_ were primarily in the cranial, anterior, and posterior directions. This might be due to motion artefacts from bowel movements and similarity in Hounsfield unit values that challenges the distinguishment of bladder from surrounding organs.

In contrast to the studies by Foroudi *et al.* [20] and Nishioka *et al*. [21], where the contours were delineated from scratch, the observer’s contours (CTV-T_ADP_) in this study were adapted from AI-generated contours. This may influence the contours and the related uncertainty. Similarly, this constitutes a possible bias compared to CTV-T_GT_, which was delineated from scratch without having the AI-contour as a start. However, it reflects the clinical situation at our as well as other institutions, where the daily CTV-T is based on an AI-generated bladder contour with manual edits [12]. Unlike the oART workflow described by Archambault *et al.* [4], our daily CTV-T is set as a copy of the (edited) AI-generated bladder contour to avoid unwanted deformations during the integrated automatic target propagation procedure. Other potential biases in CTV-T_ADP_ include awareness of participation in the study, which may affect the adapters’ delineation although they were instructed to delineate as in a clinical setting.

Our analysis revealed that AI consistently underestimated the CTV-T (Fig. 2). For patient 8, who had irregular bladder shape with small air cavities in cranial parts (probably due to tumour burden), the AI underperformed more than for other patients (Fig. 3). The evaluation of dose coverage showed that CTV-T_AI_ was generally not acceptable without editing (Fig. 5). Discrepancies between CTV-T_AI_ and CTV-T_GT_ were expected because CTV-T_AI_ was entirely CBCT-based while CTV-T_GT_ was not necessarily so. The reference CTV-T on the reference CT, used during delineation of CTV-T_GT_ and CTV-T_ADP_, was guided by a reference MRI in the caudal parts for all patients except patient 4 and 6. These discrepancies could hypothetically be accounted for by a population-based margin, potentially reducing contour editing time and making the oART workflow more time-efficient. However, not only the areas of largest deviation but also the size of the deviations in CTV-T_AI_ compared to CTV-T_GT_ varied among the patients (Supplementary Figure S2). Dose coverage was lacking in caudal areas of CTV-T_GT_ in plans optimized on PTV-T_AI_ for some patients, though not for most of them. Therefore, our data does not support concluding on a population-based margin that would be valid for the majority of patients. However, extra attention should be paid to caudal, cranial, and anterior areas of the CTV-T_AI_. The need to edit the AI-generated contours in CBCT-guided oART has been recognized in other studies. Studies reporting on bladder cancer however is lacking; a study by Azzarouali *et al.* [7] primarily report on the deformation of boosted target volume rather than the AI generated contours for oART of bladder cancer with integrated boost. Byrne *et al.* [6] report that edit of AI-generated bladder and rectum contours was done in 89 % of the sessions for prostate cancer and Bak *et al.* [11] report edit of AI-generated bladder contour in 67 % of the sessions for vulvar cancer. While both report that the level of edit usually was small, the fact that the bladder was an OAR and not a target in both studies may affect the perceived level of adjustment needed. Furthermore, as the accuracy of the AI-generated contours depend on the system’s version, improvements in future versions might affect the need for edits.

The patients for whom CTV-T_GT_ was under-covered differed for plans optimized on PTV-T_AI_ and PTV-T_ADP_. The two plans optimized on PTV-T_ADP_ that did not cover CTV-T_GT_ both belonged to patient 10 (Fig. 5). This might be explained by the lower image quality observed for this patient. By selecting a random CBCT among clinical oART fractions for this study, CBCT of good enough quality for oART was assumed. However, this was not the case for patient 10, who only received 6 oART fractions, after which it was decided to change to a non-adaptive workflow due to difficulty in distinguishing bladder from bowel in the cranial direction. It could be argued that the CBCT quality for this patient is not representative of a typical oART situation, and this could possibly warrant excluding this patient from the analysis. However, we believe it reflects the reality of implementing and performing oART clinically and thus decided not to exclude the data from the analysis. Nevertheless, this reveals a limitation of this study: the absence of image quality scoring. While we have included data on CBCT acquisition and reconstruction parameters, a scoring of image quality, as in the study by Foroudi *et al.* [20], or delineation confidence could contribute important information.

For many patients and adapters, CTV-T_ADP_ was larger than CTV-T_GT_ (Fig. 2). There was not a general over-delineation over the entire CTV-T, but rather deviations concentrated to specific areas that were patient-specific (Supplementary Figure S1). For some patients, there was a consistency over all adapters, where all CTV-T_ADP_ deviated from CTV-T_GT_ in the same area, e.g., caudally for patient 2. The consistent discrepancy caudally for patient 2 may be explained by the difficulty in distinguishing the caudal bladder border on CBCT and level of experience of the observer. It highlights the challenge with having an MR-guided delineation in CBCT-guided oART workflow and what areas to focus on in the further education of the adapters. Furthermore, it highlights possible limitations of having a single “ground truth” contour, where CTV-T_GT_ itself includes an uncertainty.

Unlike the independent and blinded delineations of CTV-T_ADP_ in this study, the adapters always work in pairs during the clinical treatments so that the daily contours are reviewed daily by a second adapter. We, furthermore, have a physician and a physicist present the first fraction, and weekly independent offline checks of contours and treatment plans by physicians and physicists. These efforts aim to reduce the inter-observer delineation variation. Multidisciplinary attendances during the first fractions provides the opportunity to discuss areas that need extra attention, either due to tumour burden and delineation difficulty, attempting to ensure the quality of contours and dose coverage of the disease. This study supports the need for such multidisciplinary efforts and continued feedback loops.

With the future aim to decide on a margin contribution of delineation uncertainty in oART, this study present novel data showing that adapters successfully re-delineate targets, supporting them as the main driver of the oART workflow. It also highlights the essential role of image quality [30]. With recent technological developments, the quality of CBCT images has significantly been improved [31]. Faster acquisition and improved reconstruction algorithms have demonstrated an image quality comparable with fan-beam CT [32]. This has the potential to reduce the delineation variation further.

In conclusion, target re-delineation in daily CBCT-guided oART of bladder cancer demonstrated non-isotropic inter-observer variation among adapters, suggesting non-isotropic margins to account for it. Manual adjustment of AI-generated contours resulted in improved accuracy in target delineation compared to ground truth and was necessary to ensure target coverage for some patients.

## CRediT authorship contribution statement

**Lina M. Åström:** Conceptualization, Data curation, Formal analysis, Investigation, Methodology, Validation, Visualization, Writing – original draft. **Patrik Sibolt:** Conceptualization, Methodology, Project administration, Supervision, Validation, Writing – review & editing. **Hannah Chamberlin:** Methodology, Software, Visualization, Writing – review & editing. **Eva Serup-Hansen:** Conceptualization, Methodology, Supervision, Writing – review & editing. **Claus E. Andersen:** Conceptualization, Methodology, Supervision, Writing – review & editing. **Marcel van Herk:** Conceptualization, Investigation, Writing – review & editing. **Lene S. Mouritsen:** Investigation, Resources, Writing – review & editing. **Marianne C. Aznar:** Conceptualization, Methodology, Writing – review & editing. **Claus P. Behrens:** Conceptualization, Methodology, Project administration, Supervision, Validation, Writing – review & editing.

## Declaration of competing interest

The authors declare that they have no known competing financial interests or personal relationships that could have appeared to influence the work reported in this paper.
