# Supplementary material for: Artificial intelligence-generated targets and inter-observer variation in online adaptive radiotherapy of bladder cancer
Source: Phys Imaging Radiat Oncol. 2024 Sep 1;31:100640. doi: 10.1016/j.phro.2024.100640 (PMC11407955; doi:10.1016/j.phro.2024.100640)
Supplement: Supplementary Data 1 [file mmc1.pdf]

Supplementary Material

Table S1. Patient characteristics and technical settings, including gender of patient, CBCT settings, CTV-T-to-PTV-T margin in cranial/caudal/anterior/posterior/left/right (cran/caud/ant/post/left/right) direction, volume of CTV-T$_{GT}$, and additional comments.

Patient	Gender	CBCT settings [kV/mA/mAs]	CTV-T-to-PTV-T margin (cran/caud/ant/post/left/right) [mm]	CTV-T$_{GT}$ volume [cm^3]	Comment
1	M	140/100/697	10/5/8/5/5/5	211.6	Nephrostomy catheter
2	F	140/100/697	8/8/7/7/7/7	60.8	Nephrostomy catheter
3	F	140/90/1456	9/7/7/7/7/7	48.7	
4	F	140/90/1456	12/8/10/10/10/8	70.1	Hip prosthesis, no ref-MR
5	F	140/100/697	12/8/10/10/8/8	51.5	
6	F	140/100/697	12/8/8/8/10/10	71.2	No ref-MR
7	M	140/100/697	10/8/10/8/8/8	88.9	
8	F	140/100/1618	12/10/10/10/8/8	104.9	Urinary catheter
9	M	140/90/1456	12/8/10/8/5/5	83.4	Prostate and seminal vesicles in CTV-T
10	F	140/90/1456	12/10/10/10/8/8	95.5	

Reference CT scans were acquired without contrast on Philips Brilliance Big Bore scanner, GE Revolution CT scanner (GE Healthcare), or Siemens SOMATOM go.Open Pro scanner (Siemens Healthineers) and reconstructed with a slice thickness of 2 mm and a pixel size of 0.93 mm x 0.93 mm or 0.98 mm x 0.98 mm. Reference MRI scans included T2-weighted sequences acquired with contrast on the Philips Panorama MRI scanner (Philips Medical Systems).

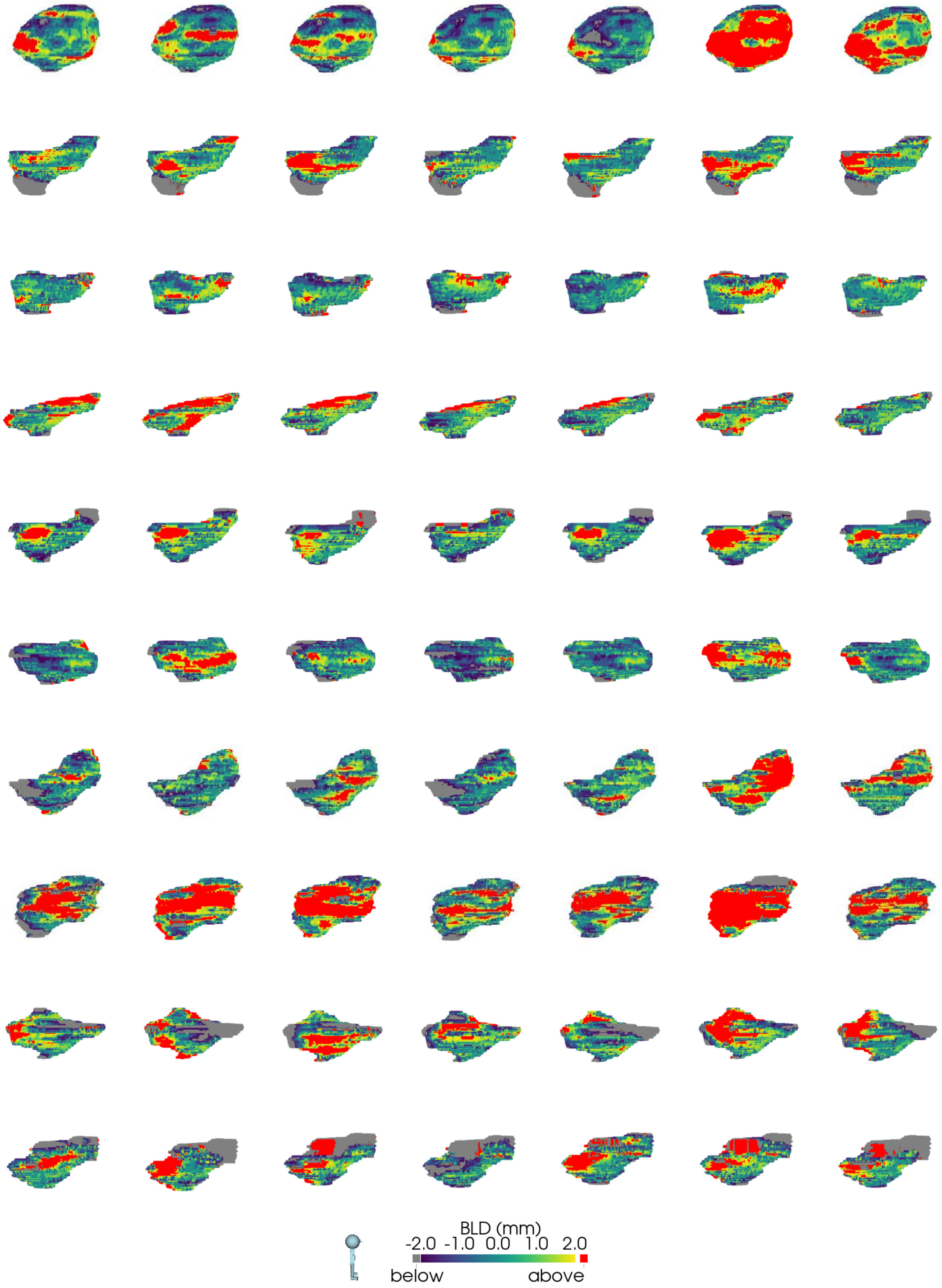

Figure S1. Viewed from the right side of the patient, i.e., anterior is to the right and posterior is to the left in each figure. 2-D colour maps of bidirectional local distances (BLD) for patient 1-10, comparing CTV-T_{GT} and CTV-T_{ADP}. Each row represents a patient, and each column represents an adapter. The colour scale goes from -2 mm to 2 mm. Values ≤2 mm are coloured in grey and values ≥2 mm are coloured in red.

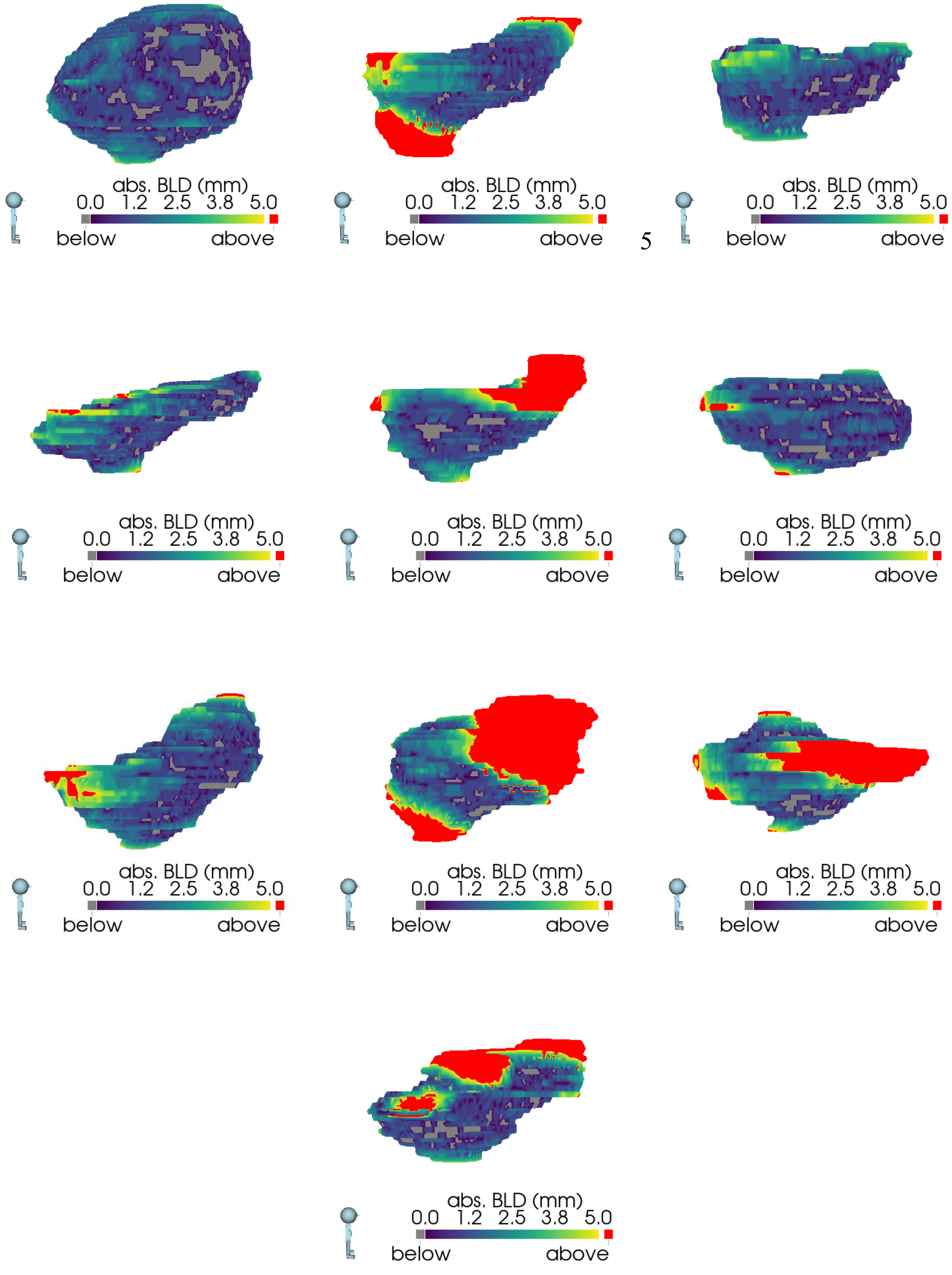

Figure S2. Viewed from the right side of the patient, i.e., anterior is to the right and posterior is to the left in each figure. 2-D colour of absolute bidirectional local distances (BLD) for patient 1-10, comparing CTV-T_{GT} and CTV-T_{AI}. The patients are ordered in a left-right, top-bottom manner. The colour scale goes from 0 mm to 5 mm, where values of 0 are coloured in grey and values ≥5 mm are coloured in red.

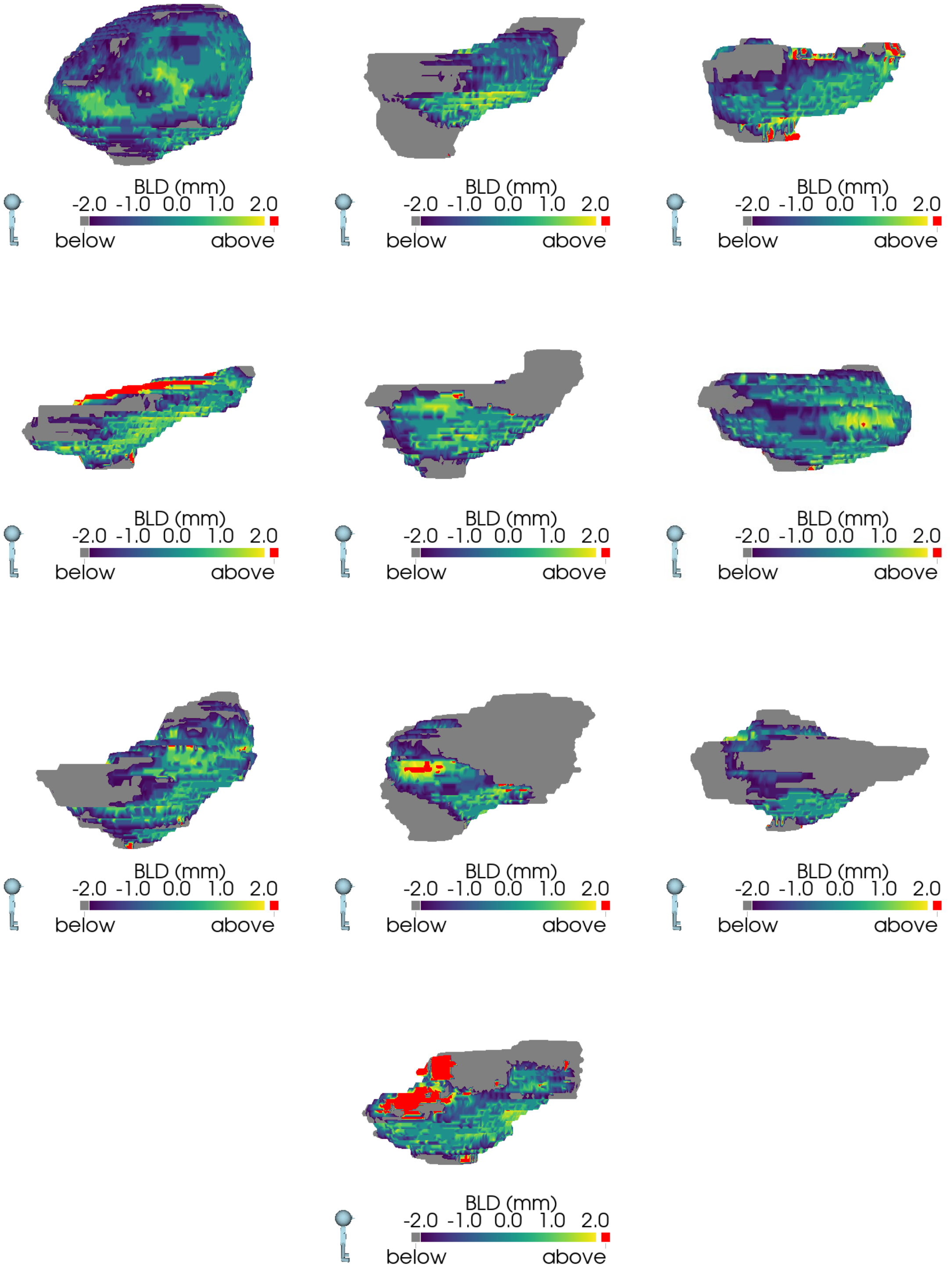

Figure S3. Viewed from the right side of the patients, i.e., anterior is to the right and posterior is to the left in each figure. 2-D colour of bidirectional local distances (BLD) for patient 1-10, comparing CTV-T$_{GT}$ and CTV-T$_{AI}$. The patients are ordered in a left-right, top-bottom manner. The colour scale goes from -2 mm to 2 mm. Values ≤2 mm are coloured in grey and values ≥2 mm are coloured in red.

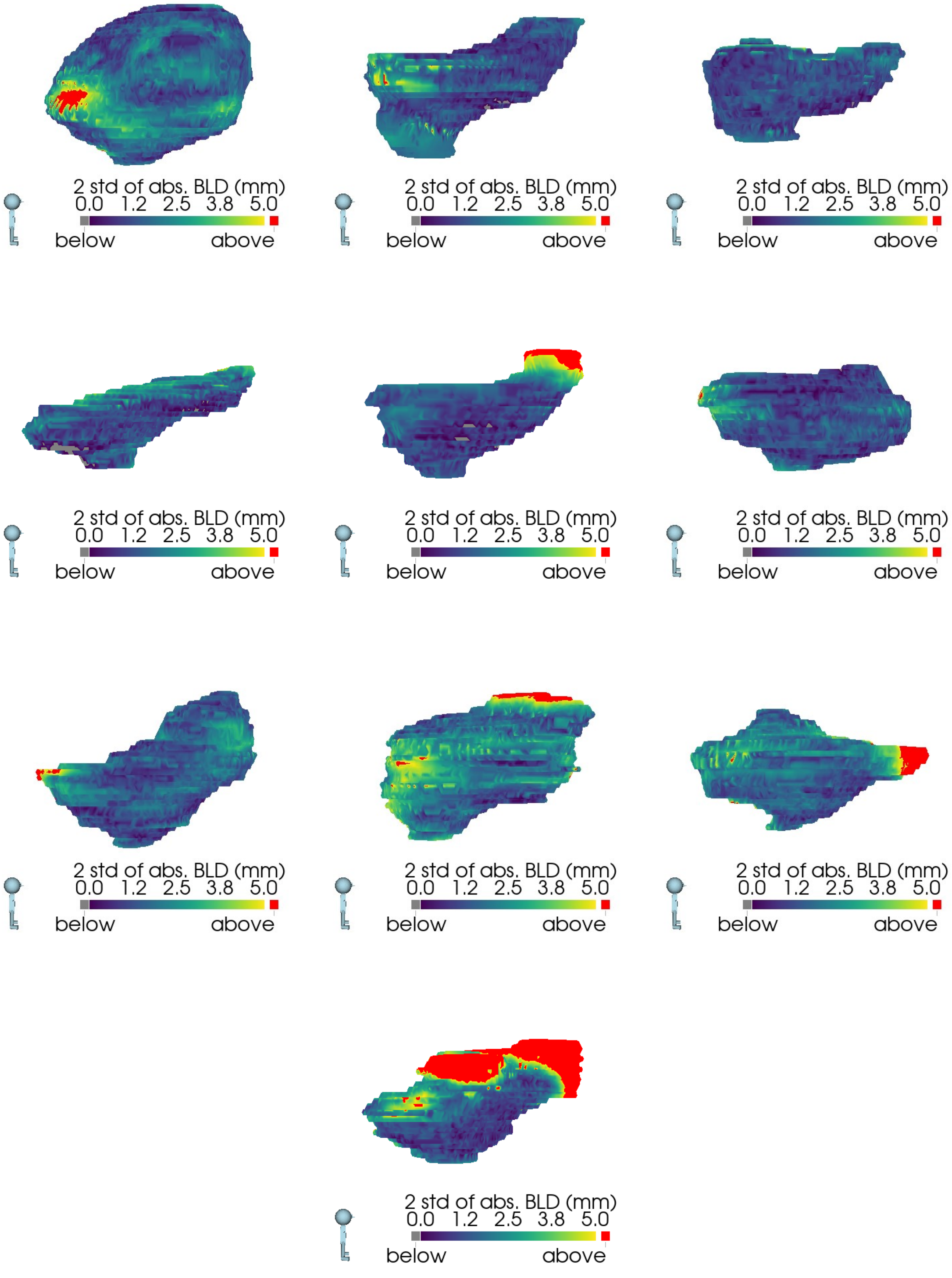

Figure S4. Viewed from the right side of the patient, i.e., anterior is to the right and posterior is to the left in each figure. 2-D colour maps of two standard deviations (std) of absolute bidirectional local distances (BLD) for patient 1-10, comparing CTV-T_{GT} and CTV-T_{ADP} across all seven adapters. The patients are ordered in a left-right, top-bottom manner. The colour scale goes from 0 mm to 5 mm, where values of 0 is coloured in grey and values ≥5 mm are coloured in red.
